# Moving to accelerated protocols of tDCS in catatonia: a case report

**DOI:** 10.3389/fpsyt.2023.1302718

**Published:** 2023-12-21

**Authors:** Noomane Bouaziz, Jean-Charles Luisada, Samir Jabri, Palmyre S.-K. Andrianisaina, Avicenne Bellis, Dominique Januel

**Affiliations:** Hôpital de Ville-Évrard, Neuilly-sur-Marne, France

**Keywords:** tDCS, ECT (electroconvulsive therapy), catatonia, brain stimulation, schizophrenia, rTMS (repetitive Transcranial Magnetic Stimulation), COVID-19

## Abstract

Catatonia is a severe and potentially life-threatening neuropsychiatric condition. Electroconvulsive therapy (ECT) is the gold standard second-line intervention for catatonia after benzodiazepine failure. However, the access to ECT can be particularly challenging, especially during periods of increased strain on medical facilities, such as the COVID-19 pandemic. Several case reports have suggested the potential efficacy of transcranial direct current stimulation (tDCS) in addressing catatonia. In our case, we present the successful application of intensive tDCS, delivering five sessions per day, each lasting 20 min, with an intensity of 2 mA. The tDCS montage involved placing the anode on the left dorsolateral prefrontal cortex (DLPFC) and the cathode on the left temporoparietal junction (TPJ). This approach was well-tolerated and resulted in a significant improvement in a 70-year-old patient with catatonia, for whom ECT was deemed necessary. While these results are promising, it is crucial to confirm them through a randomized controlled study.

## Introduction

Catatonia is a severe neuropsychiatric condition characterized by abnormal movements, behaviors, and abnormal responses to stimuli. It is a transdiagnostic syndrome which could be associated with schizophrenia, mood disorders, and some medical and addictive conditions (1).

Benzodiazepines (especially lorazepam) and electroconvulsive therapy (ECT) are the main treatments for catatonia. Given the high failure rate of benzodiazepines, especially in catatonic schizophrenia, ECT is considered the gold standard second-line treatment for catatonia (2, 3). Nevertheless, ECT remains a difficult treatment to provide, especially when reanimation facilities are under pressure, as was experienced during the coronavirus disease 2019 (COVID-19) pandemic (4).

Transcranial Direct Current Stimulation (tDCS) is a non-invasive brain stimulation method that has shown promising efficacy in the treatment of hallucinations, negative symptoms of schizophrenia, resistant depression, and substance abuse (5–7).

Several case reports have suggested that tDCS could also be a therapeutic tool for catatonia when ECT is unavailable, ineffective, or contraindicated (8).

There is currently significant interest in intensive forms of non-invasive brain stimulation (NIBS), especially after the publication of Cole and colleagues’ study, which demonstrated rapid and dramatic improvement in treatment-resistant depression through 10 daily sessions of iTBS (9). This rapid improvement could be helpful in the treatment of catatonia, given its life-threatening nature.

Here, we present the case of a patient with catatonic schizophrenia resistant to benzodiazepines, who responded favorably to accelerated tDCS, using 5 sessions a day, during the (COVID-19) pandemic when ECT was unavailable.

## Case

This case concerns a 70-year-old woman diagnosed with schizophrenia, with an unspecified onset of the illness. She is undergoing treatment with zuclopenthixol and exhibits recurrent relapses, often accompanied by catatonic features and psychotic dimensions.

In 2018, she was hospitalized for a catatonic syndrome characterized by stupor, mutism, negativism, and stereotypies. The therapeutic trial with Lorazepam was ineffective. Subsequently, a course of 10 ECT sessions was prescribed, leading to a complete disappearance of the catatonic syndrome.

During that time, a brain magnetic resonance imaging (MRI) was conducted, and the report noted poor MRI quality due to the patient’s movements, with ventricular enlargement consistent with a Scheltens score of 2. Following the resolution of the catatonic episode, the diagnosis of schizophrenia was upheld based on the presence of verbal hallucinations, delusional ideas, cognitive and behavioral disorganization. These symptoms showed improvement during this period under 20 mg of olanzapine.

After this episode, the patient was repatriated by her son to France and institutionalized in a home for the elderly.

During the 2021 COVID-19 lockdown, the patient experienced a relapse of the catatonic episode, leading to her first hospitalization at our hospital.

During this relapse, the clinical presentation was characterized by a severe catatonic syndrome featuring rigidity, waxy flexibility, maintaining bizarre postures, mutism, and complete negativism. Biochemical exploration revealed no anomalies, notably the absence of electrolyte imbalance, no biological signs of inflammation, no thyroid dysfunction, and normal levels of vitamin B12, B9, and D. Serological tests for hepatitis B, hepatitis C, HIV, and syphilis were all negative. The patient did not undergo a lumbar puncture or an electroencephalogram.

A new brain MRI was conducted, revealing no parenchymal abnormalities, no signal or morphology anomalies in the hippocampi, and an absence of significant abnormalities capable of explaining the symptomatology. Slightly enlarged lateral ventricles suggest early normal-pressure hydrocephalus.

Therapeutic trials with lorazepam up to a dose of 7.5 mg and zolpidem up to a dose of 30 mg were ineffective and have been completely and gradually stopped, as electroconvulsive therapy (ECT) has been decided upon as the treatment.

This course of ECT was conducted under general anesthesia using etomidate and neuromuscular blockade with rocuronium. Given the urgent context, electrodes were placed in a bitemporal arrangement, and titration was not performed. The initial intensity was delivered based on the age-dose formula. The patient underwent a series of 10 electroconvulsive therapy (ECT) sessions, resulting in significant clinical improvement. She was discharged in April 2021, with ongoing maintenance ECT sessions scheduled twice a month.

Unfortunately, ECT maintenance was prematurely discontinued just 1 month later. This decision was due to the increasing hospital pressure caused by the COVID-19 pandemic, which limited ECT to only curative purposes.

In July 2021, another catatonic relapse occurred and needed rehospitalization. Seven ECT sessions were performed with satisfactory but partial clinical improvement. However, due to reduced availability of anesthetists practicing ECT, the sessions were spaced out to approximately one session every 2 weeks.

Facing a severe catatonic relapse in October 2021, and because ECT was completely unavailable, the team was compelled to try another neuromodulation technique: tDCS. The patient, as well as her son who is her legal guardian, have given their consent. Ten tDCS sessions were conducted, with a frequency of 2 sessions per day, every 2 to 3 days. tDCS was delivered using a NeuroConn device (Ilmenau, GmbH) with two saline 7×5 cm electrodes. The montage used was described by Brunelin et al. (10), initially aiming to alleviate schizophrenia hallucination but which showed promising results in the treatment of catatonia (1). The anode was placed over the F3 EEG 10–20 system, thought to allow the stimulation of the left DLPFC, and the cathode was placed on the Left temporoparietal T3-P3 midpoint of the 10–20 system, thought to correspond to the left temporoparietal junction (TPJ). Stimulation was delivered with a 2 mA intensity, via 2 sessions of 20 min, spaced with a 20-min interval. This methodology is in accordance with safety guidelines on the use of tDCS (11, 12). Transcranial DCS resulted in slight improvement but with a progressive resurgence of catatonic symptoms during days without tDCS. Then treatment was optimized with an accelerated design using 5 sessions per day, spaced by a 20-min Inter-sessions interval (ISI), every day, instead of the initially prescribed 2 sessions a day.

After a total of 50 sessions (10 days of stimulation) at this accelerated pace, the catatonic symptoms significantly decreased, with the Bush-Francis scale score dropping from 36 to 13 in December 2021. The patient showed much less rigidity, echopraxia, and echolalia disappeared, and her sense of humor was retrieved. Consolidation sessions were then scheduled to amplify and maintain the clinical improvement, similar to the approach used with ECT following this pattern: 5 sessions per day, 5 times per week, then 5 sessions per day, 3 times per week, then 5 sessions per day, 2 times per week. In February 2022, after a total of 150 sessions, a complete disappearance of all catatonic symptoms was observed, with a Bush-Francis scale score of 3 (Table 1). With the confirmation of stable improvement and ongoing maintenance tDCS sessions, Mrs. B was discharged from the hospital in May 2022.

**Table 1:** Evolution of catatonia under different treatments.

Period	January 2021	February 2021	March 2021	April 2021–June 2021	July 2021	August 2021–October 2021	November 2021	December 2021	December 2021–February 2022
Brain stimulation treatment		10 ECTs sessions 2 ECTs/week	Maintenance ECTs 2 ECTs/month	**Covid-19 pressure** **ECT withdrawal**	7 ECTs sessions	**Covid-19 pressure** Irregular maintenance ECT	**Covid-19 pressure** Only 4 ECTs sessions with premature withdrawal. 10 tDCS session 2 sessions/day × 2 weeks	**Covid-19 pressure ECT unavailable** 50 tDCS 5 tDCS/day X 10 days	**Covid-19 pressure ECT unavailable** 150 tDCS with gradual decrease 5 tDCS/day × 5 days/week 5 tDCS/day × 3 days/week 5 tDCS/day × 2 days/week
Pharmacological treatment	Lorazepam trial and Zolpidem trial	Olanzapine 20 mg/day	Olanzapine 20 mg/day	Olanzapine 20 mg/day	Olanzapine 20 mg/day	Olanzapine 20 mg/day	Olanzapine 20 mg/day	Olanzapine 20 mg/day	Olanzapine 20 mg/day
Outcome	No improvement	Remission	Remission	Relapse	Improvement	Relapse	Partial improvement	Improvement	Remission
BF	40	0	0	40	3	40	36	13	3

BF, Bush-Francis Catatonia Rating Scale; ECT, Electroconvulsive therapy; tDCS, transcranial Direct Current Stimulation; Covid-19, coronavirus disease 2019 pandemic.

Once accessibility to ECT was restored, Mrs. B can again receive ECT in the event of a relapse.

It is noteworthy that tDCS was very well tolerated by the patient, and no side effects were mentioned, except for a mild and very temporary redness along the region under the electrodes.

## Discussion

This study is in accordance with Haroche et al. (1), reporting the interest of tDCS with “schizophrenia montage” in catatonia treatment. It also provides evidence for the feasibility, tolerability, and safety of repetitive sessions of accelerated tDCS in a patient with catatonia in accordance with the Mondino et al. case report (13), which inspired our stimulation methods, describing an improvement and good tolerance after two consecutive days of five tDCS sessions in highly resistant schizophrenia patient.

The high efficiency of accelerated design is probably a result of both giving a high dose of stimulation in a short time and the enhanced cumulative neurophysiological effect allowed by the optimized ISI duration. In our case, we used the same ISI duration as Mondino et al. (13) as it was suggested that the optimal ISI for tDCS should be under 30 min. Monte-Silva and colleagues (14) compared the effects of different time intervals (ISI) on the potentiation of a 13-min anodal tDCS effect. They reported that only the 20-min ISI resulted in the most robust proexcitability effect, lasting over 24 h. This effect was only mildly evident with a three-minute ISI, and it was not observed with ISIs of 3 and 24 h, or when the tDCS duration was doubled. Agboada et al. (15) reported comparable findings, with two 1 mA tDCS sessions lasting 15 min and spaced by a 30-min ISI resulting in significant effects, whereas ISIs of 60 or 120 min did not. The window of 30 min between two NIBS techniques to optimize the enhancement of the excitability after effect is also supported with TMS paired pulses (16), intermittent theta burst stimulation (17) and animal studies (18, 19).

In our study, a regimen of five transcranial direct current stimulation (tDCS) sessions per day was employed. The augmentation of the number of spaced non-invasive brain stimulation (NIBS) sessions is likely to prolong the duration of plastic changes. Nyffeler et al. (20) observed that the use of 2 continuous theta-burst stimulation (cTBS) trains resulted in a 2-h aftereffect, whereas 4 trains extended the duration to 10 h. In individuals with stroke, Cazzoli et al. (21) found that four cTBS trains improved spatial neglect symptoms for 32 h, and the application of eight trains over 2 days extended the relief to at least 3 weeks.

Finally, the most remarkable clinical result to date in using NIBS to treat depression was achieved in a randomized trial employing 10 daily sessions of iTBS (9). In addition to the inter-stimulus interval (ISI) duration, the duration of the tDCS session likely plays a crucial role in eliciting the desired neurobiological effect. A 20-min duration, as applied in our clinical case, appears to be the optimal choice. Monte-Silva et al. reported that doubling the duration from 13 to 26 min resulted in a diminished excitability effect compared to a 13-min tDCS session (14). Agboada et al. (15) reported that employing two 20 min tDCS sessions with high intensity (3 mA), while maintaining a 20-min ISI, resulted in a weaker excitability effect compared to two shorter sessions (15 min) with 1 mA intensity. Furthermore, Hassanzahraee et al. (22) reported that the excitability-enhancing effect of anodal tDCS is reversed when the duration exceeds the threshold of 26 min.

Despite the unknown exact effects of tDCS session duration and ISI mechanisms, requiring further studies, several hypotheses have been proposed to explain the prolonged effect of repeated stimulations with a 30-min inter-stimulation interval (ISI). A single stimulation would induce neuroplastic effects of the early long term potentiation (E-LTP) type by promoting the rapid phosphorylation of α-amino-3-hydroxy-5-methyl-4-isoxazolepropionic acid (AMPA) and Nmethyl-D-aspartate (NMDA) receptors and increasing their membranous expression, thereby leading to a temporary enhancement of the response to post-synaptic stimuli. On the other hand, closely spaced and repeated sessions would result in a neuronal synaptic tagging effect (15, 19), involving transcriptional and synthetic activity of AMPA, NMDA receptors, and also BDNF. This would allow for a prolonged neurological effect of the late LTP (L-LTP) type (15, 19).

Among convulsive NIBS, ECT is regarded as the second-line option following benzodiazepines and has demonstrated remarkable efficacy, even in cases where benzodiazepines prove ineffective, yielding success rates ranging from 59 to 100% (2, 8). However, notwithstanding this observed effectiveness, a meta-analysis (23) has underscored that the evidence supporting this efficacy is derived from a limited number of studies with low methodological rigor. Consequently, there is a recommendation for the initiation of a high-quality randomized controlled trial (23). Repetitive Transcranial Magnetic Stimulation (rTMS), like tDCS, is a non convulsive NIBS method. Both of them are beginning to be investigated as possible alternatives to ECT, given that they are more accessible with a better tolerance profile. To date, the effectiveness of these techniques in the treatment of catatonia has been reported only through case reports or case series. Interestingly, the reported effectiveness has been observed in catatonia, regardless of its origin—whether schizophrenic, mood-related, organic, or otherwise (1, 8, 24). Moreover, the methodologies used vary widely, targeting different brain regions with paradigms aimed at either excitatory or inhibitory effects.

The exact mechanism underlying the effectiveness of ECT, rTMS, and tDCS on catatonia remains unknown, similar to the uncertainties surrounding the pathophysiology of catatonia itself. However, based on empirical observations that antipsychotic dopaminergic antagonists worsen catatonia while benzodiazepines improve it, it has been suggested that catatonic states may be associated with a decrease in dopaminergic and GABAergic transmission (2, 3, 25). It has also been reported that catatonic states are associated with disturbances in the motor system, involving disruptions in frontoparietal networks and hyperactivity in the supplementary motor area (SMA) and pre-supplementary motor area (pre-SMA). There would also be an involvement of the autonomous, endocrine, and immune systems (3, 26).

In the existing body of knowledge, refining animal models of catatonia (3, 27) and implementing controlled clinical trials focused on Non-Invasive Brain Stimulation (NIBS) for treating catatonia is crucial. This is essential both to confirm the interest of NIBS in this indication and to explore hypotheses related to the pathogenesis of catatonia.

Currently, there are at least two randomized controlled trials (RCTs) assessing the effectiveness of rTMS in catatonia registered on ClinicalTrials.gov with the following IDs: NCT06139432 and NCT06016764.

To our knowledge, this is the first time where multiple sessions of tDCS were used on consecutive days to treat catatonia. We showed that 10 days of accelerated tDCS led to a well tolerated dramatic improvement in a patient with severe catatonia, which seemed dependent on ECT. These data are important given that tDCS is an inexpensive, portable treatment with almost no adverse effects and does not require general anesthesia. This is an important result, which can improve psychiatric conditions and catatonia in particular, which need a highly and rapidly effective treatment. Nevertheless, it has to be confirmed by a controlled trial. In fact, a very recent trial reported no superiority of 5 daily tDCS sessions over sham in the treatment of resistant depression. It is worth noting that in this trial the ISI interval was not specified (28).

## Data availability statement

The raw data supporting the conclusions of this article will be made available by the authors, without undue reservation.

## Ethics statement

Ethical approval was not required for the studies involving humans because the administration of stimulation was conducted urgently. Both the patient and her son, serving as her legal guardian, have given their consent for both the stimulation and its subsequent publication in an anonymized manner. The studies were conducted in accordance with the local legislation and institutional requirements. The participants provided their written informed consent to participate in this study. Written informed consent was obtained from next of kin of the patient for the publication of any potentially identifiable images or data included in this article.

## Author contributions

NB: Conceptualization, Investigation, Methodology, Validation, Writing – original draft, Writing – review & editing. J-CL: Writing – original draft. SJ: Investigation, Methodology, Writing – review & editing. PA: Investigation, Methodology, Writing – review & editing. AB: Writing – review & editing. DJ: Conceptualization, Validation, Writing – review & editing.
